# Programming human cell fate: overcoming challenges and unlocking potential through technological breakthroughs

**DOI:** 10.1242/dev.202300

**Published:** 2023-12-11

**Authors:** Hsiu-Chuan Lin, Aly Makhlouf, Camila Vazquez Echegaray, Dorota Zawada, Filipa Simões

**Affiliations:** ^1^ Department of Biosystems Science and Engineering, ETH Zürich, 4057 Basel, Switzerland; ^2^ MRC Laboratory of Molecular Biology, University of Cambridge, Cambridge CB2 0QH, UK; ^3^ Molecular Medicine and Gene Therapy, Lund Stem Cell Centre, Wallenberg Centre for Molecular Medicine, Lund University, 221 84 Lund, Sweden; ^4^ First Department of Medicine, Cardiology, Klinikum rechts der Isar, Technical University of Munich, School of Medicine and Health, 81675 Munich, Germany; ^5^ German Center for Cardiovascular Research (DZHK), Munich Heart Alliance, 80636 Munich, Germany; ^6^ Regenerative Medicine in Cardiovascular Diseases, First Department of Medicine, Klinikum rechts der Isar, Technical University of Munich, School of Medicine and Health, 81675 Munich, Germany; ^7^ Department of Physiology, Anatomy and Genetics, Institute of Developmental and Regenerative Medicine, University of Oxford, Oxford OX3 7TY, UK

**Keywords:** Cell programming, Genomic engineering, Human cell fate, Reprogramming, Synthetic biology

## Abstract

In recent years, there have been notable advancements in the ability to programme human cell identity, enabling us to design and manipulate cell function in a Petri dish. However, current protocols for generating target cell types often lack efficiency and precision, resulting in engineered cells that do not fully replicate the desired identity or functional output. This applies to different methods of cell programming, which face similar challenges that hinder progress and delay the achievement of a more favourable outcome. However, recent technological and analytical breakthroughs have provided us with unprecedented opportunities to advance the way we programme cell fate. The Company of Biologists’ 2023 workshop on ‘Novel Technologies for Programming Human Cell Fate’ brought together experts in human cell fate engineering and experts in single-cell genomics, manipulation and characterisation of cells on a single (sub)cellular level. Here, we summarise the main points that emerged during the workshop's themed discussions. Furthermore, we provide specific examples highlighting the current state of the field as well as its trajectory, offering insights into the potential outcomes resulting from the application of these breakthrough technologies in precisely engineering the identity and function of clinically valuable human cells.

## Teaser

**Summary:** Advances in human cell programming offer the potential to manipulate cell identity and function but face many challenges. Recent breakthrough technologies hold promise for more precise and efficient cell engineering.

## Introduction

In recent years, we have witnessed significant advancements in the field of programming human cell identity, allowing for the precise design and manipulation of cellular function within controlled environments. One aim of cell programming is to achieve specific fate outcomes tailored to the intended application, such as the generation of disease-specific cell models to study disease mechanisms (Avior et al., 2016; Trounson and DeWitt, 2016), personalised therapies (Barker et al., 2017; Zimmermannova et al., 2023), the production of cells for regenerative medicine (Vierbuchen et al., 2010; Song et al., 2012) or a deeper understanding of the underlying mechanisms governing cell fate decisions (VanHorn and Morris, 2021; Jindal et al., 2023). However, protocols for generating such desired cell types are often limited in efficiency and precision, resulting in engineered cells that fall short of fully replicating the intended identity or functional output. Such challenges are encountered across various methods of cell programming.

Cell programming approaches can be broadly categorised: (1) reprogramming, in which mature, specialised cells undergo a reversal of their developmental state, regaining pluripotency to then adopt a new cellular identity and function; (2) programming from pluripotent cells or progenitors, where less-committed cells are guided towards specific cell fates; (3) direct reprogramming or transdifferentiation, the conversion of one cell type directly into another without the need for an intermediate precursor stage; and (4) synthetic biology, which provides tools to precisely manipulate and programme cell behaviour by engineering synthetic gene circuits, modifying signalling pathways or regulating gene expression at the molecular level with unprecedented resolution.

Novel single-cell technologies hold immense potential for advancing cell and tissue engineering. They enable in-depth molecular characterisation of engineered cell states, allowing for an accurate assessment of the efficiency, precision and harmonisation of existing protocols. In addition, the availability of single-cell atlases for primary developing and adult tissues are valuable resources for guiding cell engineering efforts and predicting the necessary requirements and design strategies for generating selected cell fates. Further progress has been facilitated by CRISPR engineering and synthetic biology, enabling meticulous regulation of gene expression, therapeutic activity, and control over dosage, timing and localisation of programming factors.

The Company of Biologists organised the ‘Novel Technologies for Programming Human Cell Fate’ workshop in 2023, bringing together experts in human cell fate engineering, single-cell genomics, cell state manipulation and characterisation. The workshop provided a platform for a deeper understanding of the challenges, advancements and potential applications in programming human cell fate.

**Fig. 1.**
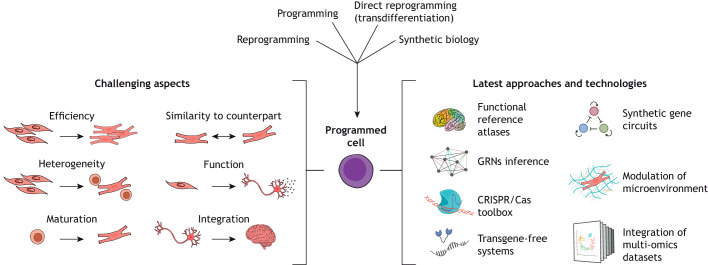
**Technological breakthroughs offer solutions to address challenges in achieving the desired cell output.** Programming human cell fate can be achieved through different approaches, including reprogramming, programming, direct reprogramming (transdifferentiation) and synthetic biology. Advances in creating comprehensive reference cell atlases and multi-omics databases, inferring gene regulatory networks (GRNs), designing precise and tunable CRISPR/Cas9-based tools, developing efficient delivery systems, refining synthetic gene circuits and recreating *in vivo* niches can help overcome barriers associated with obtaining the desired cell identity and function. These challenges include issues such as low efficiency, heterogeneity in generated cell products, limited maturation, dissimilarity to counterparts, lack of desired cell function and *in vivo* integration.

Here, we summarise the current field of human cell fate programming (Fig. 1). Drawing on insights emerging from thought-provoking discussions at the workshop, we offer a deeper understanding of the field's landscape. In addition, we provide a glimpse into the exciting prospects that lie ahead, pushing the boundaries of what can be achieved in this rapidly evolving field of research.

## Challenges

Progress in recent years makes cellular programming an exciting field. Much effort has been invested into developing engineered cells to resemble their *in vivo* counterparts, and this is already reaching clinical potential (Kirkeby et al., 2023). As exciting as this scenario appears, there are challenges and concerns that need to be addressed. Cell programming can cause unexpected outcomes (Treutlein et al., 2016) and many current approaches generate cells that recapitulate only certain aspects of their bona fide *in vivo* counterparts in terms of transcriptome, cellular properties and function. Many reasons might account for this, such as an incomplete understanding of developmental signalling cues, using a minimal set of factors to obtain desired cell types, lack of a detailed reference to guide *in vitro* work or the general artificiality of culture systems. To guarantee the safety and efficacy of cell-based interventions, it is vital to conduct comprehensive analysis and monitoring of programmed cells, through genomic profiling, functional assays and long-term observation to effectively identify and mitigate risks.

### Efficiency and heterogeneity

Efficiency in cell programming is crucial because it determines the success of programming processes while minimising off-target effects. The development of highly efficient approaches for cell conversion represents an everyday challenge, and focuses on assessing the time, scalability, reproductivity and robustness for producing specific cell types. These parameters are tested through functional *in vitro* or *in vivo* assays (e.g. transplantation experiments; Balboa et al., 2022; Kirkeby et al., 2017; Tan et al., 2018) with the goal of generating adequate numbers of programmed cells for advancing practical applications. Another constraint that influences efficiency is the presence of programming roadblocks, which involve transcriptional and chromatin features that prevent cell fate changes (Brumbaugh et al., 2019; Arabacı et al., 2021). These barriers are not covered here but are recognised as important factors that determine success when programming cells.

Heterogeneous populations in *in vitro* cell cultures can be viewed in two different ways: high cell type diversity can introduce too much ‘noise’ in the system and divert the efforts put into programming specific cell types. Therefore, controlling and minimising this effect is crucial for reliable and predictable cell reprogramming. Conversely, some scenarios require the presence of a variety of cell types for proper maturation, as is the case for complex 3D systems. Here, cellular heterogeneity is desired and advantageous (Meier et al., 2023; Drakhlis et al., 2021; Cooke et al., 2023).

Heterogeneity occurs in the source cells used for programming and in the programmed population. For the former, a major challenge is whether the choice of the source cell type or state affects the outcome. Research groups have been optimising protocols tested on different source cell types, which can possibly cause discrepancies in the results obtained and conclusions. Efforts have been invested into standardising these protocols, as well as expanding the diversity of the source cells used for programming (Karow et al., 2018; Lentini et al., 2021; Zhou et al., 2008). The extent to which diversity can be controlled is limited because cells inevitably carry pre-existing mutations. Also, molecular dynamics shape the intrinsic context of cells, which can result in certain states that are not amenable to conversion. For example, how variability in expression levels of endogenous transcription factors can determine whether cells will fail or efficiently convert cell fate (Francesconi et al., 2019).

### Maturation

A major challenge is recapitulating maturation processes for the acquisition of functional properties of mature cells *in vitro*, which is crucial for disease modelling and therapeutic translation. For this, understanding developmental maturation is essential but time, in many cases, is the limiting factor. It is necessary to speed up maturation processes that can take years *in vivo*. The artificiality of culture systems, which mainly rely on plastic dishes and daily media changes, does not recapitulate the formation and nutrition of cells and tissues in a body. Also, mature phenotype acquisition of many cells does not happen in a vacuum but is dependent on many signalling cues coming from other tissues (Cooke et al., 2023; Arterbery and Bogue, 2014). For example, in direct lineage reprogramming, the generation of a more mature phenotype could be aided by choosing an aged donor as the cell source, but that also brings other challenges regarding the overall fitness of older cells (Oh et al., 2022).

### Similarity to the primary counterpart

Nevertheless, despite the limitations of cell culture systems, we now have several cell types generated *in vitro* that resemble certain features of their *in vivo* counterparts. Conventionally, the comparison between engineered cells and *in vivo* target cells has relied on evaluating general morphologies, biomarker expressions, bulk-omics data and functional assays (Nolbrant et al., 2017; Kroon et al., 2008; Kamao et al., 2014). Recently, it has become common practice to use the transcriptome obtained *in vivo* as the blueprint to engineer the perfect cellular identity and function (Loh et al., 2014). The advent of single-cell RNA-sequencing (scRNA-seq) has revolutionised our ability to systematically and quantitatively characterise cell identities. Efforts including the Human Cell Atlas (https://www.humancellatlas.org/) and Tabula Sapiens Consortium (https://tabula-sapiens-portal.ds.czbiohub.org/) have played pivotal roles in generating comprehensive transcriptome reference atlases at single-cell resolution for diverse primary tissues, providing valuable resources for understanding cell types and states. Leveraging scRNA-seq readout, it becomes possible to quantify the transcriptome similarity between reference atlases and engineered cells, thereby facilitating the estimation of off-target lineages within the culture (La Manno et al., 2016). Though this approach is useful, it does not provide a dynamically resolved view of tissue development and it is challenging to guide *in vitro* work on snapshots that might be obtained with confounding artefacts of sample processing or with different single-cell transcriptomics methods. Although computational approaches have been developed to integrate diverse datasets, the mapping of engineered cell identities remains challenging due to differences in sequencing depth, cell clustering resolution and the absence of universally standardised cell annotations. To generate *in vivo*-like cells, we must start with a well-established *in vivo* reference to assess the validity and relevance of generated cell products and our approaches, preferably with approaches that link *in vitro* obtained properties of cells (transcriptome, proteome, function, etc.) with dynamically recorded data *in vivo* through computational modelling.

### Function

Regardless of the approach used to generate a certain cell type, it is essential that obtained cells exhibit a certain set of functions and behaviours that are characteristic for equivalent cells within the body. This is especially true for drug testing or disease modelling platforms. It seems fairly straightforward to assess cell functionality *in vitro* – whether by the measurement of action potentials for cells that are electrically active or by the measurement of proteins secreted upon stimuli for cells that are metabolically active. However, results of these measurements might be difficult to interpret: should measurements obtained on isolated cells *in vitro* be the same as cells *in vivo*? Is it possible that cells that respond to stimuli *in vitro* would fail to respond to similar stimuli *in vivo* in a timely and dose-dependent manner?

One of the best approaches to achieve the full functionality of cell products is to expose them to the *in vivo* niche through transplantation. The signalling cues coming from the microenvironment can provide the necessary stimuli, enhancing terminal differentiation or maturation (Balboa et al., 2022; Kirkeby et al., 2017). Many challenges and questions remain: is it sufficient to bring cells to a certain state of functionality and let the *in vivo* niche do the rest? Can we predict how cells will behave and mature following transplantation? Can we recreate the niche *in vitro*? Depending on the goal of the study, we might need approaches to mature cells *ex vivo*.

### Integration

Obtaining cellular products that are suitable for cell replacement is crucial not only to ensure their functional maturation but also functional integration with the surrounding microenvironment upon transplantation. The cellular behaviours and interactions within the native tissue must be considered to create an environment that supports the functionality and longevity of engineered cells. Strategies to enhance cell competitiveness including cell survival, optimising delivery methods and promoting tissue-specific interactions, can enhance functional integration.

Ultimately, ensuring the similarity of generated cells in terms of morphology, gene expression and functionality is essential to safeguard the validity and relevance of research findings, as well as the safety and efficacy of cell therapy.

## Overcoming current challenges

### Working with bona-fide reference networks for data integration

To gain a comprehensive understanding of engineered cell fates, establishing a unified framework for describing cell identities across diverse human cell atlases is crucial. The reference cell tree is a valuable approach that combines molecular states and lineage histories to address this need (Domcke and Shendure, 2023). Although single-cell genomics has transformed our understanding of cell identities, debates arise regarding whether cell identity should be solely defined by function, as cells with similar gene expression patterns may exhibit different functional behaviours (Scala et al., 2021). However, in scenarios where the *in vivo* function of cell subtypes is undefined, we contend that gene expression can serve as a bridge for extrapolating and comprehending these uncharted functions. In addition to solely relying on observed feature similarities, we can potentially disentangle cell identity emphasising functional attributes by considering cellular responses to environmental cues or perturbations (see Glossary, Box 1). Perturbation experiments and the construction of functional reference atlases can provide deeper insights into cellular functions, facilitating engineering of functional cells (Rauscher et al., 2017; http://genomecrispr.dkfz.de; https://orcs.thebiogrid.org).

**Box 1. Glossary**

**Attractor state.** The stable and well-defined endpoints of the cell engineering process, regardless of the starting conditions or the transitions that occur during the process.

**Autoencoders.** Neural network architectures used for unsupervised learning tasks that aim to encode and then decode input data with minimal error, often used in dimensionality reduction or feature learning.

**Bridge dataset (single cell).** An intermediate dataset used to connect or integrate data from different sources or experiments, often applied in single-cell analysis to infer cell states and link modalities.

**Cryo-EM.** Cryogenic electron microscopy, a technique that enables high-resolution imaging of biological molecules by freezing them in vitreous ice and visualising them using electron microscopy.

**Directed evolution.** A technique for artificially evolving proteins or other biomolecules in the laboratory and selecting desired properties or functions.

**Large language model.** A multi-layered computational model, pre-trained on very large datasets of text, that learns to recognise, predict and generate text in a given language.

**Perturbation.** The introduction of controlled disturbances or changes into a biological system in order to study its response and behaviour.

**Microfluidic controlled stem cell regionalization.** The manipulation of stem cell differentiation and positioning within microfluidic devices to control morphogen gradients, allowing the study of tissue development and organisation.

**Minimal descriptor.** A concise representation or characteristic that captures the essential information of a complex system or object.

**Molecular recording.** The process of capturing and storing molecular-level events or information within cells, often used for tracking cell lineages, cellular behaviour or environmental cues.

**Neural network.** A computational model composed of layered interconnected processing units (nodes or artificial neurons) that simulate the information processing observed in biological brains. The node layers include input, output and one or more hidden layers. Each node has an associated weight and threshold and is only activated when the output passes the threshold, passing data to the next layer.

**Neuromorphic computing.** A computing paradigm inspired by the architecture and functioning of the human brain, aimed at developing energy-efficient and parallel processing systems.

**Optimal transport (single cell).** A mathematical framework to quantify and analyse the transportation of resources from one distribution to another with minimal cost. In single-cell analysis, it is used to compare and match distributions of molecular features such as gene expression profiles.

**Organ-on-chip.** Microfluidic cell culture platforms that mimic the structure and function of human organs to study physiological responses and drug effects.

**Sketching techniques.** Methods used to approximate complex datasets with simplified representations, such as subsampling cells while maintaining rare populations, to speed up computation.

**Transformer-based models.** Advanced neural network architectures that are used in tasks involving sequential data, language understanding and generation.

It is also crucial to unravel the underlying mechanisms driving cell fate specification to effectively guide cell engineering efforts. Gene regulatory networks (GRNs) play a fundamental role in orchestrating cell fate determination, which can be inferred from gene expression patterns and relevant regulatory elements (Fleck et al., 2022; Kamimoto et al., 2023; Aibar et al., 2017; Davidson and Erwin, 2006). By comparing the GRNs of engineered cells with reference networks, we can explore whether identical fates require similar regulatory routes or if alternative expedited pathways lead to the same desired attractor states (see Glossary, Box 1). However, inferred causal relationships within GRNs often represent indirect predictions or associations. Is it possible to approach a more accurate representation of the molecular events that drive cell state transitions, thereby leveraging this knowledge to enhance cell engineering outcomes? Recent advancements in molecular recording technologies (see Glossary, Box 1) offer promising avenues for uncovering transcriptional histories and the dynamic processes underlying cell fate transitions (Farzadfard and Lu, 2018; Schmidt et al., 2018; Choi et al., 2022; Chen et al., 2022; Horns et al., 2023).

### Tools for enhancing the accuracy and precision of cell fate programming

Transcription factor-mediated cell programming remains the most widespread approach for manipulating cell fate (Takahashi and Yamanaka, 2006; Barretto et al., 2020; Missinato et al., 2023; Pierson Smela et al., 2023). Directed evolution (see Glossary, Box 1) has been used to generate new transcription factor variants with improved reprogramming speeds and efficiencies, compared with wild-type counterparts (Tan et al., 2021). However, traditional transcription factor-mediated approaches continue to raise concerns over the need to introduce exogenous genes in human cells. Alternative tools, such as CRISPR activation (CRISPRa) and CRISPR interference (CRISPRi), rely on guide RNA (gRNA)-mediated targeting of DNA, in conjunction with a deactivated Cas9 nuclease, to modulate the activity of endogenous genes (Perez-Pinera et al., 2013; Sokka et al., 2022). CRISPR-Cas13 is also a potentially versatile tool for the direct targeting and post-transcriptional regulation of RNA (Adler et al., 2022). In addition, the CRISPR-Cas12a system has the ability to process multiple CRISPR-RNAs (crRNAs) in a single CRISPR array, potentially useful for multi-gene regulation (Magnusson et al., 2021). Most cellular phenotypes, including cell states, are polygenically regulated and rely on the coordinated dosage control of multiple gene products, making multi-gene regulation a challenging yet crucial bottleneck for efficient cell fate programming. Developing robust transcriptional control systems by designing programmable gene regulatory elements with reproducible effects on the transcriptional outputs of multiple genes has remained somewhat elusive, mainly due to the cellular and genetic context-dependencies of how regulatory elements behave. Together, these tools might facilitate cell fate engineering without over-expressing exogenous genes, but they still rely on generating transgenic cells and, depending on the approach, do not necessarily confer heritable cell identities.

Transgene-free systems have emerged as promising alternatives to bypass the burden, and potential risks, of genetically modified cells. One example is the use of an antibody-based reprogramming approach, amenable to high-throughput, combinatorial library screening, which can identify cell surface-targeting antibodies with the same downstream signalling effects in the cell for reprogramming as the respective transcription factors (Blanchard et al., 2017). There is also the added benefit that antibodies have higher selectivity compared with small molecules, another popular approach for manipulating signalling pathways during cell reprogramming. Therefore, the ability to design fit-for-purpose antibodies could dramatically expand the potential of this approach. Indeed, recent advances in large language models (see Glossary, Box 1) are already seeing growing applications in generative protein design and evolution (Hie et al., 2023; Watson et al., 2023). As such, the protein design space is becoming increasingly more attainable and can serve as a ‘playground’ for generating new and functional protein-based tools for cell reprogramming. Nanobodies, for example, are increasingly applied to reprogramming immune cells for cancer immunotherapy and CAR-T cell engineering (Hie et al., 2023; Ma et al., 2020). With the potential benefits of cost, size, delivery and binding specificity compared with traditional antibodies, nanobodies may serve as a promising tool for future innovations in cell reprogramming. In addition, there are RNA-based approaches, including miRNA and mRNA (Yakubov et al., 2010; Kogut et al., 2018; Warren et al., 2010). Active areas of research aim to improve targeted cell delivery approaches (Paunovska et al., 2022), RNA encapsulation methods (Hou et al., 2021; Tanaka et al., 2023), RNA modifications that confer higher stability and lower immunogenicity (Paunovska et al., 2022; Kim et al., 2022; Liu and Wang, 2022), as well as reduce variability associated with RNA transfection efficiency (Shin and Min, 2023).

In future, advances in cell fate engineering will rely on the creation of cellular systems that can respond to spatiotemporal cues with high precision and appropriate sensitivity in a cell type-specific manner. The field of synthetic biology offers the opportunity to engineer programmable, multi-gene and multicellular systems that can potentially be constructed from modularised components, such as synthetic receptors (Morsut et al., 2016), synthetic cell-cell signalling networks (Toda et al., 2018) or synthetic, multi-stable gene circuits (Zhu et al., 2022). Such complex synthetic biological systems rely on the ability to encode complex cellular logic through genetic circuits, which have more traditionally taken inspiration from digital circuit design. More recent work has been inspired by principles of neuromorphic computing (see Glossary, Box 1; Rizik et al., 2022), by creating a neural network (see Glossary, Box 1) architecture in *Escherichia coli* to perform perceptron-based cellular computations, which can go beyond the capabilities of basic digital and analogue circuits. Together, synthetic biology tools can potentially be used for both *in vitro* applications, where precise, automated, multi-step cell fate acquisition may be needed, or *in vivo* applications, where engineered cell states that can process *in vivo* signalling cues and respond accordingly are required.

Finally, techniques that facilitate cell fate engineering by direct manipulation of the cell microenvironment, rather than the cell itself, are growing. Techniques, such as StemBond hydrogels, modulate active cell signalling pathways through the selective control of the mechanical and/or biochemical properties of the substrate. Recent work using alginate hydrogels (Elosegui-Artola et al., 2023) has shown that the viscoelastic properties of the extracellular matrix can control cell signalling, symmetry-breaking, proliferation and morphology, particularly in the context of multicellular aggregates and organoids. Complex organoid systems also rely on the co-culture of a target cell type with one or more auxiliary cell types to promote target cell fate acquisition by mechanical constraints, cell-cell signalling and growth factor secretion, which combine to create a supportive cell niche. This is exemplified by recent advances in stem cell-based models of human embryos, which precisely co-culture several defined cell types to generate higher-order complexity *in vitro* (Weatherbee et al., 2023; Ai et al., 2023; Yuan et al., 2023 preprint; Hislop et al., 2023 preprint; Pedroza et al., 2023; Oldak et al., 2023). Microfluidic approaches can also precisely control spatiotemporal signalling cues. For example, the microfluidic-controlled stem cell regionalisation (MiSTR; see Glossary, Box 1) system generates spatially patterned WNT gradients for studying neural tube development (Rifes et al., 2020). More broadly, the advent of ‘Organ-on-Chip’ systems (see Glossary, Box 1), although hampered by issues of complexity, scalability, cost and standardisation, could provide opportunities for achieving reproducible cell fate reprogramming in more chemically-defined and physically constrained microenvironments.

### Comprehensive multi-modal data integration

We previously highlighted the power of single-cell transcriptomes to describe engineered cell identities. However, to understand the mechanisms behind cell fate transitions and predict outcomes, relying solely on transcriptomics may be insufficient. Integration of diverse omics datasets could further our understanding of cell states and transitions. By combining single-cell transcriptomics with other modalities, such as chromatin accessibility, specific chromatin modifications, DNA methylation and protein measurements, we can obtain a detailed and holistic view of cellular identities (Stuart and Satija, 2019; Zhu et al., 2020; Baysoy et al., 2023). This multi-layered approach goes beyond gene expression, unravelling the intricate regulatory mechanisms that govern cell fate decisions. Incorporating protein measurements is particularly important because protein levels often deviate from gene expression levels (Vogel and Marcotte, 2012; Reimegård et al., 2021). Although direct measurement of all these modalities in single cells is not currently possible, emerging computational approaches, including integrating modalities based on a bridge dataset or optimal transport (see Glossary, Box 1), offer promising solutions (Hao et al., 2023; Klein et al., 2023 preprint). Computational efficiency has also been enhanced through sketching techniques (see Glossary, Box 1) and graphical processing unit (GPU) acceleration, enabling effective processing of atlas-scale datasets containing up to millions of cells (Hao et al., 2023; Nolet et al., 2022 preprint). Scalable single-cell multi-omics empowers us to monitor and predict outcomes following perturbations, facilitating the design of precise and effective interventions in cell engineering. This comprehensive understanding and integration of multiple omics dimensions significantly advances our capability to engineer cells with desired identities.

Single-cell spatial omics is a powerful complement to single-cell molecular multi-omics, providing valuable spatial context to molecular profiles. Technologies such as spatial transcriptomics, multiplexed protein staining, imaging mass cytometry and 3D spatial mass cytometry allow us to capture cell morphology, intracellular organisation and cellular polarity that emerge during cell-fate manipulation (Bhatia et al., 2022; Kuett et al., 2022; Rodriques et al., 2019; Gut et al., 2018; Moffitt et al., 2016; Giesen et al., 2014). Cryo-electron microscopy (Cryo-EM; see Glossary, Box 1) offers high-resolution structural information of cellular components and molecular complexes and the potential to decipher architectural changes during cell fate transitions (Pfeffer and Mahamid, 2018). Single-cell spatial omics also contribute to our understanding of cell-cell interactions and tissue microenvironments (Kanemaru et al., 2023). It enables the mapping of molecular gradients, cell-cell signalling pathways and spatially restricted niche factors that influence cell behaviour and fate decisions, which is crucial for recreating complex tissue microenvironments in engineered systems or for engineering cellular therapies that can integrate seamlessly within native tissues. For example, CellPhoneDB (Efremova et al., 2020) leverages data on the combined expression of multi-subunit ligand–receptor complexes to infer intercellular communication, and NicheNet models cell-cell communication by linking ligands to target genes (Browaeys et al., 2020). Importantly, there is now opportunity to combine intercellular communication inference tools with existing single-cell spatial omics data, integrating both molecular and spatial data to build unified, higher confidence models of functional cell-cell interactions. The common thread among these tools reflects a move towards trying to understand cell fate specification from a more holistic, multicellular context, acknowledging that cells are not solely the products of their own intrinsic molecular programmes, but also respond to their surroundings and to neighbouring cells in a cell-extrinsic manner.

There are tools to infer how single-cell GRNs respond to perturbations, with clear applications for *in silico* cell reprogramming (Kamimoto et al., 2023; Jung et al., 2021; Lotfollahi et al., 2019). Autoencoders (see Glossary, Box 1) are a deep neural network framework seeing increasing utility in the field of network biology (Theodoris et al., 2023), with the compositional perturbation autoencoder (CPA) learning to predict transcriptional perturbation responses at the single-cell level *in silico* for unseen drug dosages, cell types, time points and species (Lotfollahi et al., 2023). Of course, the question remains whether the prediction of perturbation responses on a handful of genes is sufficiently predictive of changes in cell identity. Capybara is a tool that focuses more explicitly on cell identity, exploring the continuous space of intermediate or ‘hybrid’ cell states, which are not necessarily captured *in vivo* but could still be functionally relevant in engineering cell fate transitions (Kong et al., 2022). In addition, there have been recent attempts at using transformer-based models (see Glossary, Box 1), such as the ‘single-cell bidirectional encoder representations from transformers’ (scBERT) model, which can decode and annotate both large transcriptomic and multi-omic datasets (Yang et al., 2022). As omics technologies continue to emerge, we can expect to improve our capacity to capture molecular information at cellular resolution, pertaining to the genome, epigenome, transcriptome, proteome, metabolome and molecular interactomes, among others. Importantly, computational tools will be crucial in understanding how to achieve a maximally informative but minimal descriptor (see Glossary, Box 1) of cell state, and to reduce the design space for cell reprogramming.

## Conclusions

There have been significant advancements in programming human cell identity, allowing for greater manipulation and design of cell function. Yet, challenges remain, hindering the full replication of desired cell identity and function. The Company of Biologists’ 2023 workshop on ‘Novel Technologies for Programming Human Cell Fate’ highlighted the limitations and opportunities in this field, emphasising the potential impact of recent technological breakthroughs on precisely engineering clinically valuable human cells.

There are a variety of purposes for engineering cells, from gaining a deeper developmental understanding to the clinical application of functional cell products. In each case, there will be specific limitations and outcomes, all-in-all depending on what questions we are trying to answer by programming cells.

The field of cell fate programming is rapidly advancing, there is therefore a need to achieve a unified framework to describe cell identities across diverse cell atlases. Although single-cell genomics has been transformative in understanding cell identities, there is an ongoing debate about defining cell identity solely based on gene expression profiles. It is essential to consider functional attributes and cellular responses to environmental cues. A limitation of all the technologies discussed above is the integration of approaches and datasets to uncover findings that cannot be described from a single-sided perspective. Overall, overcoming this constraint will allow us to engineer cells with desired identities and improve our ability to read and manipulate molecular information at a more holistic level. Continued advancements in multi-omics technologies and computational tools combined with the latest cell culture techniques will be crucial in shaping the future of the field.
